# Development of a Comprehensive Lesion Severity Classification Model for Largemouth Bass (*Micropterus salmoides*) Ranavirus (LMBV) Based on Machine Vision

**DOI:** 10.3390/ijms26188810

**Published:** 2025-09-10

**Authors:** Hui Sun, Jixiang Hua, Yifan Tao, Ziying Yang, Taide Zhu, Siqi Lu, Wen Wang, Yalun Dong, Linbing Zhang, Jixiang He, Jie He, Jun Qiang

**Affiliations:** 1 Wuxi Fisheries College, Nanjing Agricultural University, Wuxi 214081, China; sunhhh0126@126.com (H.S.); huajixiang@foxmail.com (J.H.); yangziying021@163.com (Z.Y.); qran0829@gmail.com (T.Z.); linda1995king@163.com (W.W.); rspy259695@163.com (L.Z.); 2 Key Laboratory of Freshwater Fisheries and Germplasm Resources Utilization, Ministry of Agriculture and Rural Affairs, Freshwater Fisheries Research Center, Chinese Academy of Fishery Sciences, Wuxi 214081, China; taoyifan@ffrc.cn (Y.T.); lusiqi@ffrc.cn (S.L.); dongyalun@ffrc.cn (Y.D.); 3 Fisheries Research Institute, Anhui Academy of Agricultural Sciences, Hefei 230041, China; hejixiangah@sina.com

**Keywords:** *Micropterus salmoides*, LMBV, damage classification, machine vision

## Abstract

This study presents the development of a quantitative evaluation method utilizing machine vision technology to characterize the extent of body surface damage in largemouth bass (*Micropterus salmoides*) infected with largemouth bass ranavirus (LMBV). High-resolution, multi-angle images (6000 × 4000 pixels) of the body surface from 239 infected specimens were acquired at a fixed distance of 40 cm using a SONY ILCE-7RM3 digital camera within a GODOX-LST60 softbox. Key parameters, including the number of segmented injury areas, the count of body surface lesions, and the total lesion area, were analyzed. These parameters were integrated through principal component analysis (PCA) to construct a comprehensive damage scoring model. The severity of viral-induced body surface damage was categorized into four grades: uninjured (0), minor injury (1), moderate injury (2), and severe injury (3). Histopathological examination revealed that early-stage infection (grade 1) predominantly exhibited localized hemorrhagic spots in the muscular region of the body side (B/E region) with limited lesion area. In contrast, moderate to severe infections (grades 2–3) were characterized by extensive ulceration, muscle necrosis, and visceral lesions, including hepatic fibrosis and splenic granulomatous formations. Quantitative real-time PCR (qRT-PCR) analysis demonstrated a progressive upregulation of pro-inflammatory cytokines (*IL-6*, *IL-8*, *TNF-α*, *CXCL2*) in immune organs, concomitant with increased expression of apoptosis-related genes (*CASP8*, *CYC*). This study successfully established a rapid and objective quantitative grading system for ranavirus infection, offering a novel technical approach for early diagnosis and precise prevention and control strategies against largemouth bass ranavirus.

## 1. Introduction

As the global population’s demand for high-quality animal protein continues to rise, fish have emerged as a vital source of nutrition, recognized for their high-quality proteins, unsaturated fatty acids, and diverse micronutrients, rendering them among the healthiest dietary options [1]. The China Aquatic Products Report 2025 shows that China’s total aquatic products amounted to 74.1 million tons, thereby maintaining a dominant position in the global aquaculture sector [2]. Among these, largemouth bass (*Micropterus salmoides*) has experienced significant growth as a key commercial fish species in China. Originally native to California, USA [3], largemouth bass was introduced to China in the 1980s and has since become a prominent freshwater aquaculture species due to its favorable attributes, including palatable flesh and rapid growth rates [4]. The recently published China Fisheries Statistical Yearbook (2024) [5], indicates that largemouth bass aquaculture production reached 888,000 tons, reflecting an 11% year-on-year increase and ranking seventh among freshwater fish species in China [5]. However, with the rapid development of the largemouth bass aquaculture industry, a series of problems have arisen, among which disease problems are particularly prominent [6], causing a loss rate of more than 20% [7]. Common disease problems include parasitic diseases [8], such as rotifers, melioidosis and sporozoites; bacterial diseases [9], such as nocardia and gill rot; and viral diseases [10,11], such as ranaviruses and elasmobranchs. Among these, ranavirus disease is the most difficult to control and the most lethal fish disease under intensive aquaculture conditions.

Largemouth bass ranavirus (LMBV), classified within the genus *Ranavirus* in the family Iridoviridae [12], was initially identified and reported in China in 2009 in association with largemouth bass ulcer syndrome [13]. Ranavirus exhibits diverse transmission pathways, rapid spreading, and wide dissemination, with infection mortality rates reaching up to 60% during outbreaks [14,15,16,17]. In advanced stages of severe infection, affected fish develop cutaneous ulcers alongside pathological manifestations such as infectious necrosis of the spleen and kidneys, often resulting in widespread mortality. LMBV proliferation is most active within the temperature range of 24–30 °C [18], a temperature range similar to the optimal growth environment of largemouth bass, leading to a high incidence of disease during culture [19]. To enhance resistance to LMBV and elucidate the underlying pathogenic mechanisms, precise and objective methodologies are critical for quantifying alterations in pathological phenotypes. Currently, injury assessment in LMBV-infected fish predominantly relies on subjective visual inspection of lesion prevalence or molecular assays targeting specific gene fragments. However, conventional evaluation techniques are dependent on empirical observation [20] and exhibit a false-negative rate exceeding 50%. Laboratory methods of quantitative PCR (qRT-PCR) [21] have high specificity but require specialized equipment and cannot be monitored in real time. Consequently, in view of these limitations, there is an urgent demand for the development of novel methodologies that provide enhanced accuracy and reproducibility, enabling objective and quantitative evaluation of LMBV infection severity in largemouth bass, thereby facilitating the formulation of effective disease management strategies.

With the rapid advancement of artificial intelligence technologies, intelligent aquaculture has emerged as a sustainable trend toward increased automation and intelligence within the contemporary aquaculture industry [22]. Among these technologies, machine vision (MV) stands out as an automated and cost-efficient tool [23] that has demonstrated considerable potential in aquaculture applications. MV enables the digitization of infected organs and phenotypic manifestations to extract phenotypic data, and it has achieved notable progress in the detection and quantification of disease symptoms [24]. Recent advances in deep learning algorithms applied to MV systems, particularly focusing on fish species, have revealed that MV represents one of the most versatile and effective approaches in image analysis [25,26], with widespread utilization across various aquaculture monitoring domains. MV-based methodologies have been successfully implemented in multiple fish species [27], such as spleen phenotyping after visceral white nodule disease (VWND) infection in large yellow croaker (*Larimichthys crocea*) [28], monitoring of abnormal behaviors in farmed large yellow croaker [29], and quantification of multiple types of diseases in crucian carp (*Carassius auratus*) [30]. Nevertheless, LMBV, a viral disease characterized by progressive pathological changes, currently lacks standardized criteria for injury assessment and rapid evaluation tools. In order to be able to better quantify disease variability among diseases, this study was based on MV acquisition of injury images of LMBV-infected largemouth bass, which were screened to 239 largemouth bass based on the characteristics of body lesions, and the three indexes, namely, number of segmented injury regions, number and area of injury, were quantified into a single index by high-resolution multiangle image acquisition, and combined with principal component analysis (PCA) to integrate the multidimensional indexes. A comprehensive injury scoring model was constructed to classify the degree of injury into four grades: uninjured (0), minor (1), moderate (2) and severe (3). The model’s reliability was validated through histopathological examination and qRT-PCR, thereby providing a scientific foundation for the effective management of largemouth bass diseases.

## 2. Results

### 2.1. Measurement of Growth Indicators in Virus Infection Breeding Experiments

**Figure 1:**
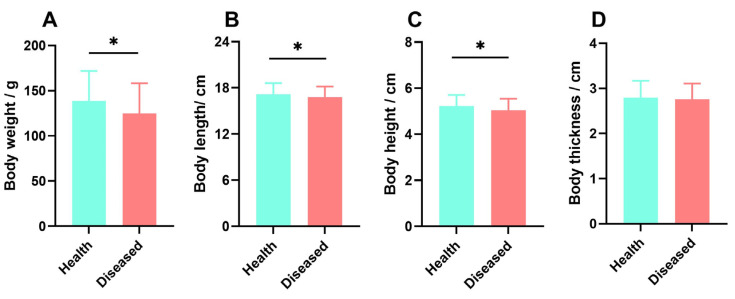
Comparison of growth data between healthy and diseased largemouth bass individuals, including: (**A**): Body weight comparison between healthy and diseased largemouth bass individuals; (**B**): Body length comparison between healthy and diseased largemouth bass individuals; (**C**): Body height comparison between healthy and diseased largemouth bass individuals; (**D**): Body thickness comparison between healthy and diseased largemouth bass individuals, Where * Indicates a significant difference between the two groups, *p* < 0.05.

Following the conclusion of the aquaculture experiment, the surviving individuals in the concrete pond were counted and examined. The mortality rate was 40.4%, with a total of 310 surviving largemouth bass. After testing with the kit, 71 healthy individuals were identified, with an infection rate of 86.3%. Based on external physical characteristics, individuals with lesions such as spotted ulcers, red hemorrhagic spots, and swollen gills and cheeks were identified. Ultimately, 239 individuals with external physical damage were selected for subsequent damage grading assessment. The growth data for the two groups are shown in Figure 1. The body weight (124.93 ± 33.27 g), body length (16.79 ± 1.37 cm), and body height (5.04 ± 0.50 cm) of the largemouth bass in the infected group were significantly lower than those of the healthy group (*p* < 0.05), while the body thickness was not significantly different (*p* > 0.05).

### 2.2. Collection and Analysis of Body Surface Injury Image Data

**Figure 2:**
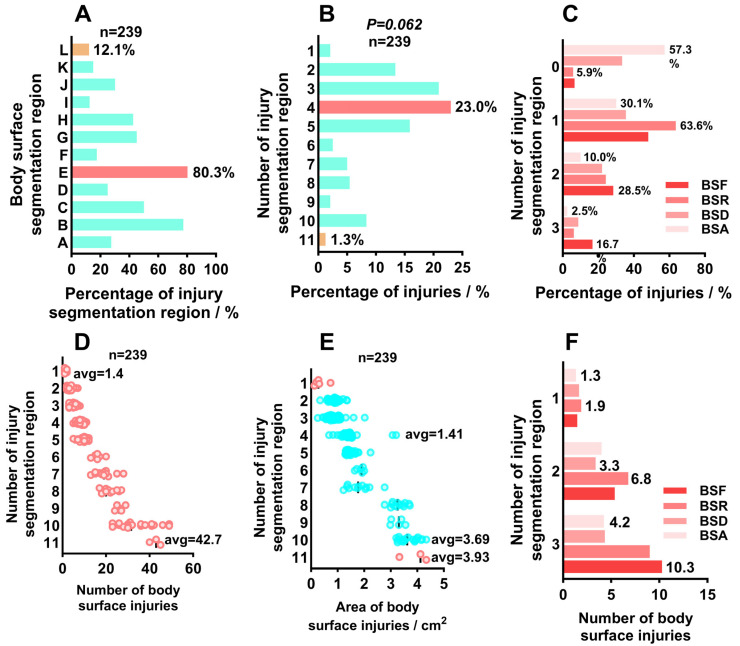
Surface injury image data: (**A**): The probability of injury occurring in individuals within 12 injury segmentation zones; (**B**): An analysis of the distribution of injury areas; (**C**): The probability of 0, 1, 2, and 3 injury segmentation areas in the front, back, abdomen, and back of the body surface; (**D**): The relationship between the injury segmentation area and the average number of injuries on the body surface of individuals; (**E**): The relationship between the injured area and the average number of injuries on the body surface of individuals; (**F**): The number of injuries corresponding to the four injury segmentation areas of the front, back, abdomen, and back of the body surface, Where the boxes represent individual data points, and the circles represent the mean values.

Damage phenotype data were collected from 239 individuals with external body damage, revealing a distinct regional specificity in damage distribution (Figure 2). First, the frequency of damage occurrence in 12 different segmentation zones was calculated for the 239 individuals with external body damage, as shown in Figure 2A. Zones E and B had the highest damage rates, reaching 80.3% and 77.4%, respectively, while Zones L, I, and K had the lowest injury frequencies (less than 15%), indicating that the virus was more likely to infect the muscle tissues on both sides of the fish body (*p* < 0.01). Further statistics were conducted on the number of injury-affected segmentation zones per individual to evaluate the extent of injury spread. The analysis of injury zone distribution numbers (Figure 2B) showed that, as depicted in Figure 2B, the highest number of individuals with four injury regions on the body surface was 55, accounting for 23.0%, while the number of individuals with three injury regions was also as high as 50. The lowest number of individuals with 11 injury regions was only 3, and the number of individuals with only one injury region on the body surface was also low, at only 5. The number of injury segmentation zones in 239 individuals followed a normal distribution (*p* = 0.062). The results show that the highest frequency occurs when one injury segment is present on each of the four body surfaces, with the highest frequency on the dorsal surface at 63.6% and the lowest frequency on the ventral surface at 30.1%. The frequencies of three and zero damaged segments on the four body surfaces were both low. The highest frequency of three damaged segments on the dorsal surface was 16.7%, while the highest frequency of no damage on the ventral surface was 57.3% (Figure 2C). The results indicate that iriditis infection is more likely to cause damage in the important muscular trunk regions of the body surface.

The ImageJ (v1.8.0) results are shown in Figure 2D. When the number of injury segmentation zones was 11, the average number of surface injuries per individual reached 42.7. As the number of segmentation zones decreased, the number of injuries decreased significantly, with individuals having only 1 segmentation zone averaging only 1.4 injuries (Figure 2D). In terms of injured area, the average injured area of individuals with 11 segmentation areas was 3.93 cm^2^, that of individuals with 10 segmentation areas was 3.69 cm^2^, and that of individuals with a maximum of 4 segmentation areas decreased to 1.41 cm^2^ (Figure 2E), indicating that the injured area of a single region decreased as the number of segmentation areas decreased. Further analysis of injury distribution across the front, back, dorsal, and ventral regions of the body surface revealed that the number of injuries increased with the number of injury partition zones (Figure 2F). Among these, the front region had the highest average number of injuries at 10.3, while the ventral region had the lowest at 1.3, consistent with the viral spread patterns observed in the injury partition zones.

### 2.3. Analysis of Surface Injury Characteristics

**Figure 3:**
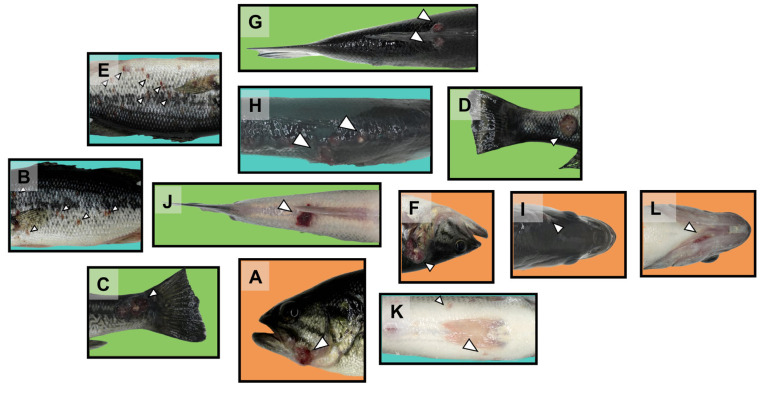
Surface characteristics of ranavirus infection in 12 injury segmentation zones, where white capital letters (**A**–**L**) indicate the 12 specific anatomical regions defined in Figure 3. White arrows point to examples of red hemorrhagic spots.

An analysis of typical pathological symptoms in the 12 divided regions of the four body surfaces (Figure 3)—the front, reverse, dorsal, and abdominal surfaces—revealed significant differences in pathological manifestations between different regions. In regions B and E, which had the highest frequency of injury, high-density red hemorrhagic spots appeared on the body surface, but there were few ulcerative lesions. Areas C, D, G, and J in the tail stalk showed characteristic muscle necrosis, accompanied by scale loss and large areas of ulcerative lesions, and the affected areas were generally accompanied by significant redness, swelling, and bleeding. In region H, large areas of tissue necrosis also occurred at the dorsal fin, and red or white nodules appeared. The virus spread over a large area, and once damage occurred in this region, the spread of the virus and the extent of the lesions were most significant. In contrast, the head region (regions A, E, I, and L) had a lower frequency of damage, but the gill cover area was more prone to pathological changes such as redness and ulceration. The abdominal K region exhibited the least severe damage, with only small, scattered red hemorrhagic spots.

### 2.4. Establishment of a Comprehensive Injury Scoring Model

**Figure 4:**
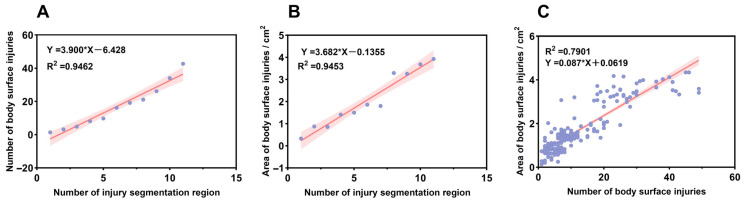
Correlation analysis between three variables: number of injury segmentation regions, number of body surface injuries, and areas of body surface injuries: (**A**): Correlation analysis between the number of injury segmentation regions and the number of body surface injuries; (**B**): Correlation analysis between the number of injury segmentation regions and areas of body surface injuries; (**C**): Correlation analysis between the number of body surface injuries and the areas of body surface injuries.

Preliminary results indicate a high correlation between damage segmentation area, damage count, and damage area. Pearson correlation coefficients were used to quantify the correlations among these three variables. As shown in Figure 4, with damage segmentation area as the independent variable and damage count and damage area as the dependent variables, the correlation coefficients were 0.9462 and 0.9453, respectively, indicating a very strong positive correlation. Similarly, the correlation coefficient between the number of injuries and the injured area is also high, at 0.7901, indicating that there is significant redundancy between the three variables. Therefore, this experiment used PCA to reduce the dimensions of these three variables, construct a single indicator that can comprehensively quantify the degree of surface injury, and establish injury grading criteria.

After standardising the number of injury segmentation regions, the number of surface injuries, and the areas of surface injuries, the PCA dimension reduction results are shown in Table 1. PC1 explains 72.3% of the variance, satisfying the dimension reduction requirements. By calculating the covariance matrix and eigenvalue decomposition, the principal component load of PC1 is obtained (Table 2), and PC1 is defined as the comprehensive lesion score.

Based on the PC1 loading score, a formula for calculating the comprehensive lesion score is constructed:

$$\mathrm{comprehensive}\;\mathrm{lesion}\;\mathrm{score}=0.59\times N_{1}+0.63\times N_{2}+0.68\times S$$

Taking a random sample as an example (the number of injury segmentation regions is 7, Min-Max normalization is 0.7, the number of surface injuries is 8, the linear translation z-score is normalized to 0.7, the injured area is 0.689 cm^2^, and the quadratic normalization is 0.11), the standardized comprehensive lesion score is as follows: 0.59 × 0.7 + 0.63 × 0.7 + 0.68 × 0.11 = 0.93. After calculating the comprehensive lesion scores of 239 largemouth bass, the values were sorted from high to low, and the comprehensive lesion scores were divided into three levels using the quantile method:

0 < comprehensive lesion score≤0.53 for minor injuries;
0.53 < comprehensive lesion score≤1.13 for moderate injuries;
1.13 < comprehensive lesion score≤3.65 for severe injuries.

**Figure 5:**
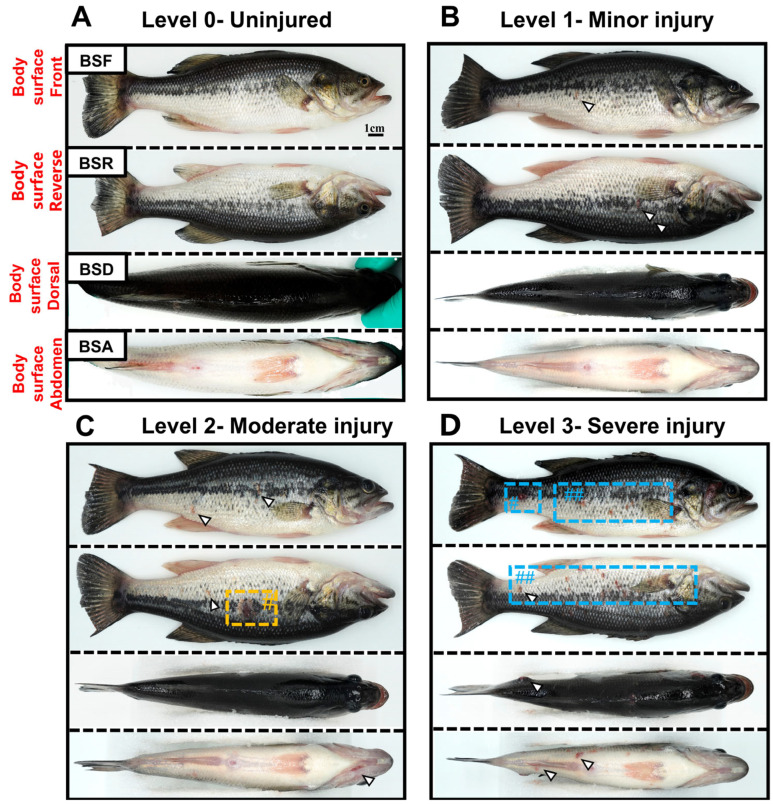
Damage grading criteria for largemouth bass infected with LMBV: (**A**): Level-0, (**B**): Level-1, (**C**): Level-2, (**D**): and Level-3, where # indicates the severity of damage and the white arrow pointing to an example of a red bleeding spot. Yellow and blue boxes highlight representative surface ulceration areas at the moderate and severe stages, respectively.

Based on the comprehensive injury score, this study established a set of criteria for determining the severity of external injuries caused by largemouth bass ranavirus infection and drew a diagram illustrating the severity of injuries (Figure 5). The specific classification is as follows:

Individuals with intact body surfaces and no visible damage after being diagnosed with ranavirus infection are classified as the undamaged grade, denoted by 0. Individuals with a small number of red hemorrhagic spots on the front and back of the body surface and no obvious ulcerative lesions are classified as minor injured, represented by 1. Individuals with large areas of ulceration on the body surface and obvious red hemorrhagic spots on the gill cover, caudal peduncle and other parts are classified as moderately injured, represented by 2. Individuals with extensive red hemorrhagic spots on the body surface, accompanied by ulcerative lesions and necrosis of the body surface muscles, are classified as severely injured, represented by 3.

### 2.5. Histopathological Analysis

**Figure 6:**
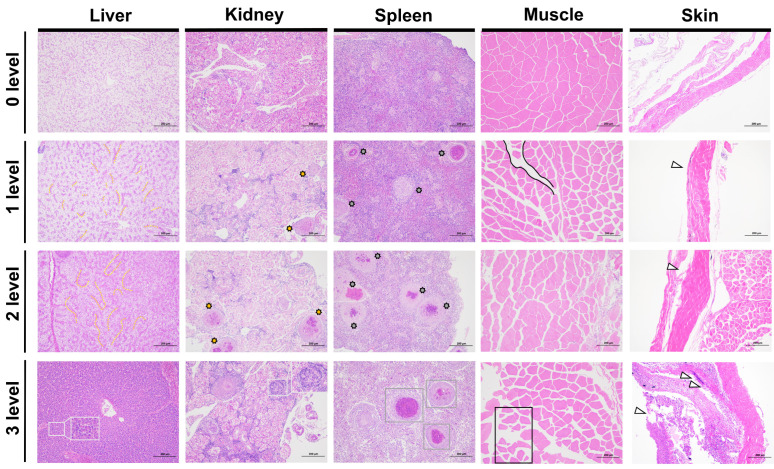
Damage grading criteria for largemouth bass infected with LMBV. Liver: Pathology progresses from focal hepatocyte necrosis to extensive fibrosis and pseudolobule formation. Kidney: Lesions evolve from vacuolar degeneration of renal tubular epithelium to extensive necrosis and granulomatous proliferation. Spleen: Characterized by lymphocyte proliferation and granuloma formation. Muscle: Progresses from vacuolization of muscle fibers to fragmentation, dissolution, and collagen hyperplasia. Skin: Demonstrates transition from collagen proliferation in the dermis to basement membrane edema and inflammatory infiltration, Where yellow dashed lines indicate areas of focal hepatocyte necrosis; white rectangles represent enlarged views of granulomas; gray rectangles, yellow and gray irregular circles denote increasing number and size of granulomas; black lines and rectangles highlight vacuolization in muscle tissue; white triangles point to localized pathological foci in skin tissue.

Based on the four injury grades classified by the comprehensive lesion score model, we compared the pathological sections of different tissues of largemouth bass infected with LMBV (Figure 6). The progressive pathological changes in each tissue are summarized in Table S1 to facilitate clearer comparison and identification of key histological features across different injury grades. The comparison revealed that all tissues exhibited varying degrees of progressive pathological damage as the comprehensive injury score increased.

In liver tissue: At Level-0, the liver is enclosed within a fibrous connective tissue capsule, without obvious lobulation. The hepatic lobule structure is intact, hepatocytes exhibit regular morphology, and the cytoplasm is uniform, with no signs of necrosis or inflammatory infiltration. At Level-1, focal hepatocyte necrosis is observed, accompanied by loose cytoplasm, oedema, and increased staining in some cells. At Level-2, nuclear condensation worsens, with nuclear fragmentation and dissolution, and varying numbers of inflammatory cells infiltrating. At Level-3, infiltration worsens further, with widespread proliferation of fibrous tissue dividing the hepatic lobules and enveloping them into irregularly shaped hepatic cell clusters of varying sizes, forming pseudo-lobular structures.

In kidney tissue: At Level 0, normal glomerular and tubular structures can be observed, with no abnormalities in the interstitium. At Level-1, tubular epithelial cells exhibit vacuolar degeneration. At Level-2, the density of inflammatory cell infiltration in the interstitium increases compared to the uninfected group. The number of granulomas increases synchronously with splenic lesions. At Level-3, there is extensive tubular shedding, widespread tissue structural necrosis, and an increased number of granulomas.

In spleen tissue: At Level-0, splenic corpuscle structure is clear, and the ratio of white pulp to red pulp is normal. At Level-1, splenic corpuscles begin to decrease, white pulp and lymphocytes increase, and a small amount of granuloma formation occurs. At Level-2, multiple granulomas increase, which is positively correlated with the degree of infection. Red pulp is congested, and macrophages accumulate. At Level-3, there is widespread granulomatous inflammation, spleen tissue structure disorder, and necrosis in some regions.

In muscle tissue: At Level 0, muscle fibres are neatly arranged with no degeneration or inflammation. At Level-1, myoplasmic vacuolisation occurs. As the infection worsens, at Level-2, inflammatory cell infiltration and widening of the intramuscular membrane oedema occur. At Level-3, the severe infection stage, muscle fibres rupture and dissolve, with local collagen fibre proliferation.

In the skin: At Level-0, the collagen fibres in the dermis are loosely arranged, and the epidermis is tightly connected to the dermis. At Level-1, collagen fibres in the dermis proliferate, and the normal loose connective tissue spaces disappear. At Level-2, the basement membrane zone at the junction of the epidermis and dermis becomes oedematous, with the appearance of a homogeneous, lightly stained transparent zone. At Level-3, vacuolar degeneration occurs in the cytoplasm, and inflammatory reactions such as lymphocyte and macrophage infiltration appear around the blood vessels in the dermis.

During the LMBV infection process, the kidneys and spleen are damaged relatively quickly and are the most severely affected organs.

### 2.6. qRT-PCR Results

**Figure 7:**
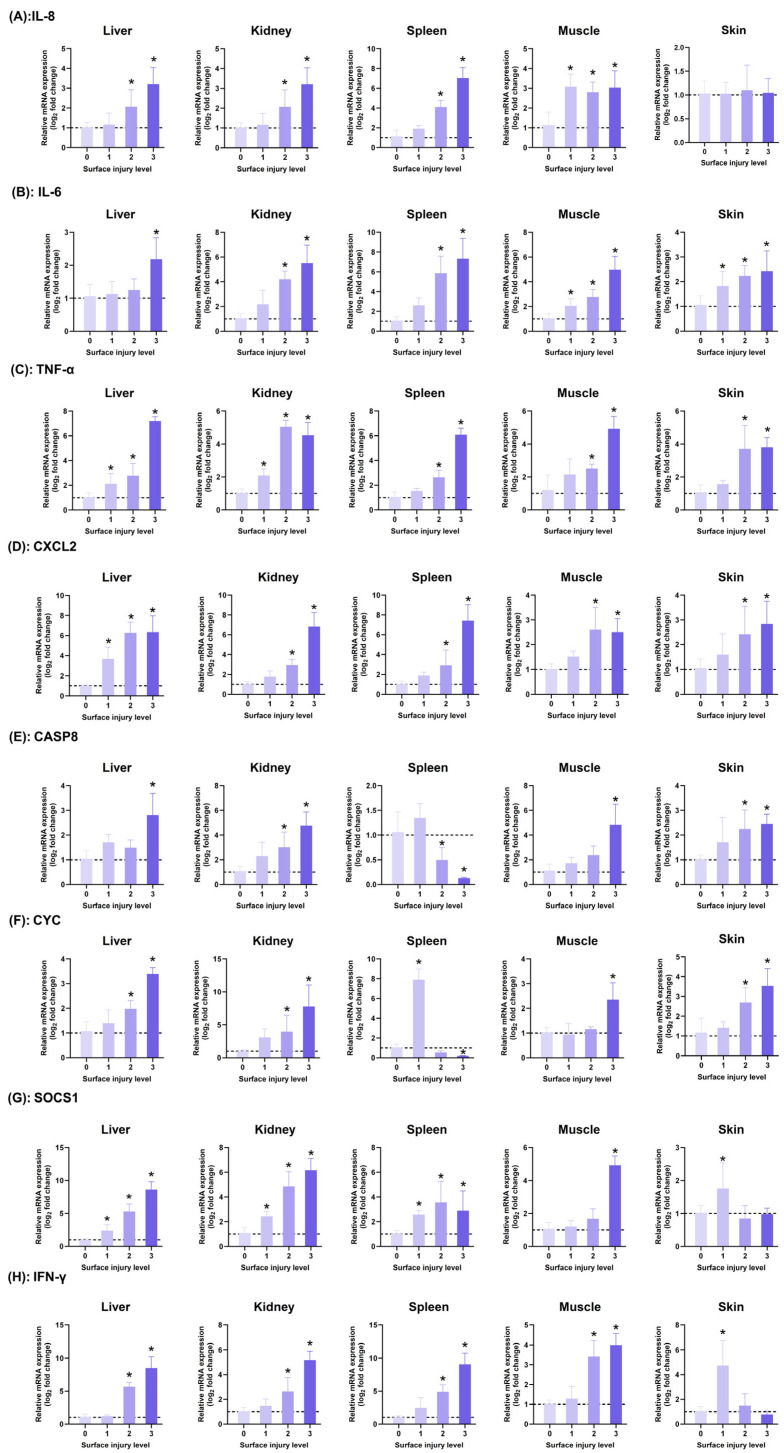
Relative mRNA expression levels of genes at different infection levels in the liver, spleen, kidneys, muscles, and skin of largemouth bass infected with LMBV: (**A**): *IL-8*, (**B**): *IL-6*, (**C**): *TNF-α*, (**D**): *CXCL2*, (**E**): *CASP8*, (**F**): *CYC*, (**G**): *SOCS1*, and (H): *IFN-γ*. * Indicates significant differences between the uninjured (0) group and different severity groups after LMBV infection (*p* < 0.05) (n = 9), Where the darker the rectangle color, the higher the injury level. The dashed line indicates a vertical coordinate value of one.

Based on the four injury grades classified by the comprehensive lesion score model, we systematically analyzed the expression patterns of immune-related genes and found that the host immune response showed obvious grade-dependent changes as the comprehensive lesion score increased (Figure 7).

At Level-0, all detected organizations showed baseline expression levels for eight genes, including pro-inflammatory factors (*IL-6*, *IL-8*, *TNF-α*, *CXCL2*) and apoptosis-related genes (*CASP8*, *CYC*).

At Level-1, all genes were upregulated, with *TNF*-α, *CXCL2*, and *SOCS1* showing significantly increased expression in the liver (*p* < 0.05), and *TNF*-α and *SOCS1* showing significantly increased expression in the kidneys (*p* < 0.05); *CYC* expression was significantly increased in the spleen (*p* < 0.05) and reached the peak value among the four levels. In muscle tissue, *IL-8* expression increased to 2.7 times that of Level-0, and in the skin, *IL-6*, *SOCS1*, and *IFN*-γ were significantly upregulated (*p* < 0.05).

At Level-2, most of the tested tissues showed significant changes: pro-inflammatory factor expression continued to increase, with *IL-6* expression in the spleen reaching 5.5 times that at Level-0 (*p* < 0.05), and *CXCL2* expression in the kidneys reaching 1.7 times that at Level-1 and 2.8 times that at Level-0. The expression of the apoptosis-related gene *CYC* in the liver was 1.8 times higher than at Level 0 (*p* < 0.05). *SOCS1* and *IFN-γ* were significantly upregulated in the liver, kidney, and spleen (*p* < 0.05), while their expression in the skin was not significantly different from the control group (*p* > 0.05). *CASP8* and *CYC* expression decreased in the spleen.

**Figure 8:**
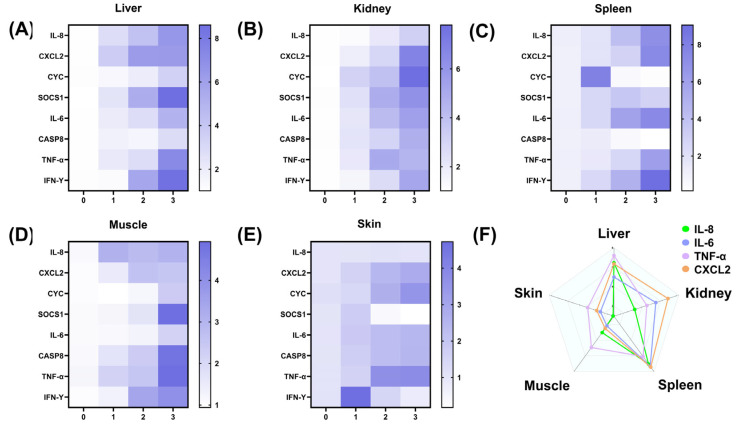
Heat map of relative mRNA expression levels of *IL-6*, *CXCL2*, *TNF-α*, *CASP8*, *SOCS1*, *CYC*, *IL-8*, and *IFN-γ* in largemouth bass following LMBV infection, and expression patterns of inflammatory genes during severe infection, including: (**A**): Heat map of expression in liver, (**B**): Heat map of expression in kidney, (**C**): Heat map of expression in spleen, (**D**): Heat map of expression in muscle, (**E**): Heat map of expression in skin, (**F**): mRNA expression levels of *IL-8*, *IL-6*, *TNF-α*, and *CXCL2* during severe infection.

At Level-3, gene expression exhibited a polarized pattern: pro-inflammatory factors remained highly expressed in the liver, spleen, and kidneys (*IL-6* reached a peak of 6.8-fold in the spleen, *p* < 0.05), *CASP8* and *CYC* expression levels are low in the spleen, apoptosis-related genes reach peak expression levels in muscle tissue (*CASP8* increases to 4.3-fold), *SOCS1* expression in skin tissue is not significantly different from the control group (*p* > 0.05), and *IFN-γ* expression in the liver increases to 8.5-fold compared to Level-0. Notably, during the severe injury stage, gene expression also exhibited tissue-specific differences: in the liver (Figure 8A), *SOCS1* (8.6-fold) and *IFN-γ* (8.5-fold) expression was highest; in the kidney (Figure 8B), *CYC* (7.7-fold) and *CXCL2* (6.8-fold) showed the highest expression levels; in the spleen (Figure 8C), *IFN-γ* (9.0-fold) and *CXCL2* (7.4-fold) showed the highest expression levels; and in the muscle and skin (Figure 8D,E), gene expression levels were generally lower than those in visceral organs (*p* < 0.05).

When diseased largemouth bass were at a severe injury level, the mRNA expression levels of inflammatory genes *IL-8*, *IL-6*, *TNF-α*, and *CXCL2* were compared across different tissues (Figure 8F). It was found that the expression levels of these four genes were higher in liver, spleen, and kidney tissues than in muscle and skin. Among them, *IL-8* and *TNF-α* had the highest expression levels in spleen tissue and the lowest in skin; *IL-6* and *CXCL2* also exhibited the highest expression levels in the spleen, followed by the kidney and liver, with muscle and skin showing similar expression levels, which were the lowest.

## 3. Discussion

### 3.1. Methodological Innovations in Machine Vision Technology for LMBV Diagnosis

Machine vision is a technique that employs machines to substitute for human visual assessment in measurement and evaluation tasks, thereby substantially improving production flexibility and automation [30]. In the present study, MV was utilized to perform automated quantitative analysis of surface damage on largemouth bass specimens, addressing the limitations of subjective manual observation and the inefficiency inherent in conventional laboratory testing. Images of the body surfaces from 239 infected individuals were collected and analyzed in conjunction with principal component analysis (PCA) to develop a comprehensive lesion scoring model aimed at objective classification of viral infection severity. The comprehensive lesion score model integrates three principal indicators: the number of damaged regions, the number of lesions, and the total area of surface injuries, thereby mitigating the bias associated with single-parameter analyses. PCA results revealed that the injured area carried the greatest weight (loading = 0.68), underscoring its critical role in evaluating disease severity. This finding aligns with pathological observations of extensive ulcerative lesions on the body surface during advanced stages of largemouth bass virus (LMBV) infection [31], suggesting that lesion area may serve as a direct indicator of viral replication intensity. This observation is consistent with prior studies demonstrating a positive correlation between lesion size and viral load in mammalian herpesvirus infections [32,33]. In contrast, the number of lesions (loading = 0.63) and the number of lesion regions (loading = 0.59) correspond to the density and spatial distribution of viral dissemination, respectively. The relatively lower weight of the lesion region count may reflect the restricted spatial distribution of lesions in early infection stages, given LMBV’s pronounced tropism for the liver and spleen [34]. Moreover, extensive lesions localized within a single region may exert greater lethality than smaller lesions dispersed across multiple regions. By integrating multidimensional indicators, the comprehensive lesion scoring model overcomes the limitations of traditional single-indicator approaches. While such multiparametric assessment methods have been extensively applied in human medicine—for instance, in immunohistochemical quantitative analyses of tumor pathology [35]—their utilization in aquatic disease research remains limited. This model not only enhances the scientific rigor and objectivity of lesion grading but also provides empirical data to inform the development of targeted prevention and control strategies. In practical terms, varying injury grades can guide differential intervention measures, thereby optimizing reductions in mortality rates and associated economic losses.

This investigation represents the inaugural utilization of machine vision (MV) for the quantitative evaluation of early-stage largemouth bass virus (LMBV) infection, introducing a novel methodology for diagnosing aquatic diseases. Recent advancements in aquaculture research have increasingly employed this technology, predominantly concentrating on the preliminary diagnosis of fish diseases, behavioral monitoring, and disease surveillance [30]. MV has been applied across various fish species, including the detection of white nodule disease in large yellow croaker (*Larimichthys crocea*) [29], analysis of largemouth bass feeding behavior, and the development of early warning systems for Nocardia infections [36,37]. Through the analysis of surface images from 239 infected specimens, it was observed that individuals with early, mild infection primarily exhibited localized lesions accompanied by minor hemorrhaging, suggesting that the virus was in an initial phase of localized replication [38]. During this infection stage, the affected regions in largemouth bass predominantly appeared as localized lesions within the muscle tissue along the lateral sides of the body, which progressively expanded to adjacent areas as the disease advanced; notably, lesions were infrequently found in regions with higher adipose content, such as the abdomen. This phenomenon may be due to the fact that the sides of the fish are more susceptible to pathogens or friction damage during the farming process, and the ranavirus preferentially attacks the muscle tissue on the sides of the body, while the fat-rich abdomen may inhibit the spread of the virus through anti-inflammatory factors [39]. These findings align with model validation results, which indicate that early LMBV infection in largemouth bass elicits localized inflammatory responses, stimulating immune cells to secrete pro-inflammatory cytokines (e.g., *IL-6* and *IL-8*), thereby inflicting substantial damage to the skin and muscle. Notably, during the severe injury phase, gene expression also exhibits tissue-specific differences: *SOCS1* and *IFN-γ* expression peaks in the liver, potentially reflecting a robust antiviral state or immune dysregulation in the primary target organ for viral replication. This pattern is a common feature across many fish viral infections. For instance, in grass carp reovirus (GCRV) infection, a dramatic upregulation of pro-inflammatory cytokines such as *TNF-α* and IFN was similarly observed and considered a key factor contributing to host tissue injury [40]. The significantly elevated *IFN-γ* expression observed in the liver in this study mirrors findings in rainbow trout infected with Viral Hemorrhagic Septicemia Virus (VHSV) [41], underscoring the central role of the type I interferon system in the anti-fish viral response. However, unlike certain viruses such as Spring Viremia of Carp virus (SVCV) that primarily induce intense inflammatory responses [42], LMBV infection laterally exhibited reduced *CXCL2* expression and downregulation of apoptosis genes (*CASP8*, *CYC*) in the spleen. This suppression of immune responses may be associated with immune evasion mechanisms employed by the virus. Studies indicate that Decapod Irisvirus 1 (DIV1) infection in shrimp affects the TNF pathway [43], while Singapore Grouper Irisvirus SGIV specifically binds to and inhibits the STING protein, thereby blocking the host’s TNF-mediated signaling pathway [44]. LMBV may employ a similar mechanism, suppressing the final defense response in the spleen, a critical immune organ. Furthermore, during viral infection, upregulation of *SOCS1*—a key negative regulator of the JAK/STAT signaling pathway—typically serves as a host feedback mechanism to prevent excessive inflammation [45,46]. This study observed significant *SOCS1* upregulation during LMBV infection, particularly in the liver. This may represent a failed attempt to control persistent inflammation or result from the virus actively manipulating host signaling pathways to facilitate its own replication. These comparative analyses indicate that despite shared immune activation pathways, each host–virus interaction exhibits unique characteristics, necessitating further functional studies on LMBV’s immune evasion strategies.

### 3.2. Analysis of the Correlation Between Surface Lesion Characteristics and Immunopathology

Histological evaluation constitutes a crucial tool for disease diagnosis, offering a direct indication of the extent of pathogen infiltration within host tissues [47]. In this study, pathological observations were integrated with quantitative gene expression analysis, a methodology extensively employed in the evaluation of histopathological alterations in fish species [48,49]. The findings demonstrated that the classification outcomes derived from the comprehensive lesion score model exhibited strong concordance with both histopathological assessments and immune-related gene expression profiles, thereby substantiating the significant association between the severity of external body surface damage and lesions in internal organs. Specifically, an increase in the comprehensive lesion score corresponded with a progressive exacerbation of histopathological damage in critical immune organs, including the liver, spleen, and kidneys, alongside a concomitant upregulation of pro-inflammatory cytokines and apoptosis-associated gene expression. This observed correlation between external and internal pathological manifestations provides a robust scientific foundation for the utilization of the comprehensive lesion score model in the diagnostic evaluation of LMBV infection.

In the minor injury stage (total lesion score ≤ 0.53), the primary clinical presentation on the body surface consisted of localized hemorrhagic spots predominantly distributed in the lateral muscle region (B/E area). It should be specifically noted that ‘early infection’, as defined in this study, refers exclusively to the clinical infection stage characterized by the earliest, mild lesions detectable via machine vision on the body surface. Although the virus has already replicated within the body and activated an immune response at this stage, pathological injury to visceral organs remains mild and focal, far from progressing to severe, extensive necrosis or fibrosis. Histological examination revealed that, during this initial phase, cell necrosis was confined to localized areas within the liver and spleen, accompanied by a modest upregulation of pro-inflammatory cytokines such as *IL-6* and *IL-8*. These findings suggest that the virus was in the early stages of replication. This observation aligns with the results reported by Xu et al. [35], who identified increased expression of pro-inflammatory factors in largemouth bass following LMBV infection as a hallmark of NF-κB/MAPK pathway activation [50,51], indicative of the host’s immune response to infection. As the infection worsened, individuals with moderate injury (0.53 < composite lesion score ≤ 1.13) developed large areas of surface ulcers, accompanied by redness and swelling of the gill cover and caudal peduncle. Histopathological analysis at this stage demonstrated a marked increase in inflammatory cell infiltration within the spleen and kidneys, alongside upregulation of apoptosis-related genes such as *CASP8* and *CYC*, suggesting active host immune regulation to contain viral dissemination. Notably, muscle tissue emerged as a frequent site of lesion development during LMBV infection, with early-stage muscle fiber vacuolization evolving into muscle dissolution and collagen fiber hyperplasia as the disease advanced [52]. This finding also suggests that the high incidence of lesions in the lateral muscle region may be related to the virus’s special affinity for muscle tissue, but the specific mechanism needs further study. In the severe injury stage (combined lesion score > 1.13), extensive ulceration and muscle necrosis were evident on the body surface, accompanied by profound structural damage to the liver, spleen, and kidneys, characterized by fibrosis and pseudo-lobule formation. Quantitative RT-PCR analyses revealed peak expression levels of pro-inflammatory mediators such as *TNF-α* and *CXCL2* in immune organs; however, *CXCL2* expression in the spleen exhibited a slight decline during the late phase of severe infection, possibly reflecting viral immune evasion strategies. The study further demonstrated that ranavirus infection not only induces overt inflammatory damage on the body surface but also impairs host growth and development. At this time, qRT-PCR and histopathological data indicated significant injury to metabolically critical organs, including the liver and intestines, accompanied by sustained elevation of inflammatory cytokines (e.g., *IL-6*, *IL-8*). This phenomenon may be attributable to the antiviral immune response prioritizing amino acid allocation toward immune functions, thereby suppressing growth-related pathways such as IGF-1. Comparable findings have been documented in the gills of grass carp (*Ctenopharyngodon idella*) [53] and the intestines of black carp (*Megalobrama amblycephala*) [54], indicating attenuated inflammatory responses. At this juncture, the majority of fish exhibited severe surface damage, which may be associated with compromised barrier function. Furthermore, during severe infection, decreased *CXCL2* expression in the spleen and downregulation of apoptosis-related genes suggest that LMBV may facilitate immune evasion by suppressing host defense mechanisms. Collectively, these findings substantiate the rationality of the proposed infection grading system.

One of the core objectives of this study was to establish an MV model capable of objectively quantifying the severity of LMBV infection. As shown in Table S2, the injury levels defined by this model (Level 0–3) demonstrated high consistency with intrinsic histopathological severity and the intensity of systemic immune responses. This not only validates the efficacy of the MV technique but, more importantly, directly links these quantifiable surface “digital phenotypes” to the host’s internal “molecular phenotypes.” This demonstrates that the comprehensive injury score provided by MV serves as a non-invasive, reliable indicator for predicting the host’s internal immune status and pathological processes. This holds significant practical value in aquaculture settings where molecular testing is impractical. The comprehensive injury scoring model developed in this study is presently limited to the assessment of LMBV infection in largemouth bass. Its applicability across different fish species for evaluating LMBV infection has yet to be established. Moreover, the precision of conventional image-processing techniques for injury detection in complex aquaculture settings requires further enhancement. This study has achieved quantitative analysis of body surface injuries based on the ImageJ platform. However, the current image segmentation method relies on manual annotation. This process may introduce observer bias, particularly when injury margins are indistinct or colors resemble healthy tissue. To minimize human error, image quality was standardized during acquisition by maintaining consistent shooting distance, lighting conditions, and camera settings. All annotations were performed by a single experienced operator during segmentation, with multiple review mechanisms ensuring data consistency. Furthermore, the injury region classification system developed in this study relies on clear anatomical landmarks, further reducing interference from subjective judgment. Although image acquisition and segmentation in the current study still rely on manual operations and have not yet been automated, the primary objective of this research is to complete a proof-of-concept demonstration. This involves verifying that machine vision technology can objectively quantify skin lesions caused by LMBV infection and demonstrating that these quantitative results show significant consistency with histopathological changes and immune gene expression levels. This provides a crucial theoretical basis and data support for developing a rapid, objective, and non-invasive diagnostic tool for LMBV infection. Future research may integrate deep learning algorithms such as convolutional neural networks (CNNs) to enhance the accuracy and efficiency of injury identification. These advancements will facilitate the early detection of LMBV-infected largemouth bass, enabling timely intervention to prevent disease progression and mitigate associated losses.

## 4. Materials and Methods

### 4.1. Sample Collection

The F_3_ generation largemouth bass population obtained from the Freshwater Fisheries Research Center of the Chinese Academy of Fisheries Sciences was used for the experiment. Before the experiment, random samples of the population were tested (n = 30) to confirm that no LMBV was carried. Before the formal experiment, the experimental fish were temporarily reared in a 4.5 × 8.8 × 1 m indoor culture tank for 2 weeks to adapt to the culture environment, during which the water temperature was maintained at 26 ± 0.5 °C, dissolved oxygen > 6 mg/L, and pH 7.2–7.6. At the beginning of the experiment, 600 largemouth bass with robust bodies, normal feeding, and similar specifications (body weight: 21 ± 0.5 g; body length: 12.3 ± 0.8 cm) were selected and transferred to a 2 × 8.8 × 1 m indoor culture tank. Largemouth bass were transferred to a 2 × 4 × 1 m cement pool, in which the water was filtered, disinfected, and fully aerated.

### 4.2. Isolation and Culture of LMBV Strains

Largemouth bass with natural onset of the disease during pre-culture were collected, and individuals with physical characteristics consistent with ranavirus disease were selected, including extensive ulceration of the body surface, swollen and ulcerated fin bases, and whitish livers, blackened spleens, and enlarged kidneys upon autopsy. Tissue homogenates were taken from the liver, spleen, and kidney under aseptic conditions and mixed for virus identification and isolation, purification, and culture. The samples were tested by ranavirus Nucleic Acid Test Kit (Guangzhou Double Helix Gene Technology Co., Ltd., Guangzhou, China) to confirm that the individuals were infected with ranavirus.

The homogenate of the disease samples was freeze-thawed three times and centrifuged at 4000 rpm for 30 min at 4 °C. The supernatant was extracted and filtered through a 0.22 μm filter, and the filtrate was stored at −80 °C. Carp epithelial tumor cells (EPC) [55] were used to culture LMBV. A total of 200 μL of filtrate was mixed with 800 μL of M199 culture medium and inoculated with healthy EPC and adsorbed at 25 °C for 1 h. The cells were supplemented with 4 mL of M199 culture medium containing 2% FBS, and then, after 1 h, placed in a cell culture incubator containing 5% CO_2_ at 25 °C until 80% of the cell monolayers showed lesions. Cells were subjected to three cycles of freezing and thawing, and then centrifuged to obtain the supernatant of the target virus. Virus titers (TCID50) were calculated using the Reed–Muench [56] method, and the supernatant containing the target virus was collected. Based on the cytopathic effect of LMBV on EPC, the TCID50 of the virus samples was determined to be 4 × 10^5^/mL. The purified viruses were stored at −80 °C until use.

### 4.3. Viral Infection Farming Tests

A total of 600 experimental fish were prepared, and 20 experimental fish were randomly selected for LMBV pathogen screening before the infection experiment. The experimental fish were tested using the ranavirus nucleic acid detection kit (Guangzhou Double Helix Gene Technology Co., Ltd., Guangzhou, China) to confirm that there was no infection. The experiment was started after confirming the absence of infection. The submerged method of virus infection was used to simulate the natural infection, and a 4-week pre-test was conducted to determine the appropriate concentration of virus infection. Sixty healthy largemouth bass (weight 21 ± 2 g) were selected and randomly divided into four groups (n = 15) and exposed to LMBV concentrations of 10, 20, 40 and 80 TCID50/mL. The time of death and number of deaths per day were recorded during the period, and the infection concentration was confirmed after 4 weeks based on the results. Pre-experiment through daily observation found that the cumulative infection rate of the 10 TCID50/mL group was less than 30% at 8 weeks, which could not effectively establish the disease model; the 80 TCID50/mL group showed 60% acute mortality at week 3, which did not meet the conditions of the study of progressive infection; and the 40 TCID50/mL group presented a 50–60% infection rate at week 4, and the development of body lesions was regular, so it was determined to use the lower level of infection concentration of 40.0 TCID50/mL as the final infection concentration for largemouth bass.

The virus solution was evenly splashed in the culture tank (2 × 4 × 1 m) with 800 mL of virus solution to maintain the water concentration at 40.0 TCID50/mL. During the experimental period, 2/3 of the culture water was replaced every week and re-sprayed with the virus solution and replenished with about 267 mL of virus solution, and the virus titer of the water body was monitored regularly to keep it unchanged. The culture environment was strictly controlled in the following parameters: water temperature 26 ± 0.5 °C, dissolved oxygen ≥7.5 mg/L, and total ammonia nitrogen ≤ 1 mg/L. Feed was fed regularly at 08:00 and 16:30 every day at a rate of 3–5% of body weight. The whole infection cycle lasted for 8 weeks, during which the clinical symptoms of the fish were observed and recorded every day, and the number of deaths was counted at the end of the experiment, and the survival of healthy largemouth bass and the number of infections were confirmed by the ranavirus nucleic acid detection kit (Guangzhou Double Helix Gene Technology Co., Ltd., Guangzhou, China). Typical lesions such as spots and ulcers, red hemorrhagic dots or red and swollen cheeks were used as criteria to select the experimental fish for image acquisition.

### 4.4. Measurement of Growth Indicators

At the end of the infection culture experiment, the experimental fish were lightly anesthetized with 200 mg/L MS-222, and then the following growth indexes were standardized after the excess water was gently absorbed from the body surface with sterile filter paper: final body weight was measured with a precision electronic balance (accuracy 0.01 g), and the following morphology parameters were measured with digital calipers (accuracy 0.01 cm): (1) total length: straight line distance from the end of the muzzle to the end of the caudal fin; (2) body height: vertical distance from the anterior base of the dorsal fin to the base of the ventral fin; (3) body thickness: horizontal distance between the left and right sides of the widest part of the body The following morphological parameters were measured using digital calipers (0.01 cm accuracy): (1) total length: the straight line distance from the tip of the muzzle to the end of the caudal fin; (2) body height: the vertical distance from the anterior base of the dorsal fin to the base of the ventral fin; and (3) body thickness: the horizontal distance between the left and right sides at the widest point of the fish. All measurements were made on a standard laboratory bench, ensuring that the measurement plane was horizontal and the line of sight for reading was perpendicular to the measuring scale.

### 4.5. Machine Vision-Assisted Injury Data Collection

A SONY ILCE-7RM3 (Sony Corporation, Tokyo, Japan) digital camera (75 mm fixed-focus lens, F/11 aperture, 1/200 sec shutter speed) was used to collect multi-angle images of the body surface frontal, reversal, dorsal, and abdominal of 239 LMBV-infected individuals in a small soft-lighting camera box (GODOX-LST60; LST-60, Godox Photo Equipment Co., Ltd., Shenzhen, China), with a distance from the acquisition lens to the flat surface of about 40 cm. In order to harvest complete phenological data of individuals, four complete body surface phenotypic images of largemouth bass (LMBV), body surface frontal, body surface reverse, body surface dorsal, and body surface abdominal, were captured, and a total of more than 1500 injury image counts were obtained.

### 4.6. Analysis of Injury Data

A method for quantitative analysis of body surface injury was established using ImageJ (v1.8.0): a weighted average method [57] was used to transform the image into an 8-bit grayscale map with the following formula:

$$Gray=\frac{R\times 30+G\times 59+B\times 11}{100}$$

where *R*, *G*, and *B* represent the pixel values of the red, green, and blue channels, respectively.

**Figure 9:**
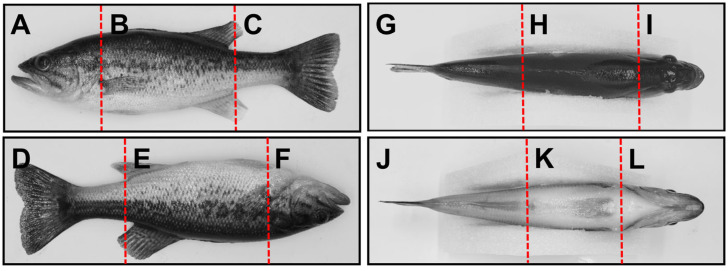
Schematic representation of the 12 segmented regions of the body surface of the largemouth bass.

Subsequently, the body surface was divided into 12 injury regions (A–L) according to the anatomical characteristics (Figure 9) of the body surface frontal, body surface reverse, body surface dorsal and body surface ventral. The frontal surface of the body was divided into zones A (anterior end of the mandible to the posterior edge of the gill cover), B (posterior edge of the gill cover to the end of the anal fin) and C (end of the anal fin to the end of the caudal fin); the reverse side of the body was divided into zones D (end of the caudal fin to the end of the anal fin), E (end of the anal fin to the posterior edge of the gill cover), and F (posterior edge of the gill cover to the anterior end of the mandible); and the dorsal region was divided into zones G (end of the caudal fin to the anterior end of the second dorsal fin), H (anterior end of the dorsal fin to the posterior end of the skull) and zone I (posterior end of the skull to the anterior end of the lower jaw); the ventral region was divided into zones J (end of the caudal fin to the vent), K (vent to the posterior edge of the gill cover) and L (posterior edge of the gill cover to the anterior end of the lower jaw). After defining the scale distance according to the scale using ImageJ (1.8.0), the area of the injury region was finely labeled using the lasso tool to quantify the number of body surface injuries and the area of the injury in each injury region, and to count the number of injury regions of each injured individual for the construction of the subsequent evaluation system of the injury rating.

### 4.7. Establishment of Criteria for Determining the Level of Injury

The number of segmented regions, number of injuries and area of injuries on the body surface of all injured individuals were counted, and the three variables were normalized in order to eliminate the difference in magnitude of the differences between these three different variables. The Min-Max normalization method was used for the number of segmented areas:

$$X^{\prime }=\frac{(X-Xmin)}{(Xmax-Xmin)}$$

where *X* is the value of the segmented region and *Xmin* and *Xmax* are the minimum and maximum values of the segmented region, respectively.

The continuous variables, number of injuries and area of injuries, were standardized using the z-score method:

$$Z=\frac{(X-\mu )}{\sigma }$$

where *X* is the number of injuries or area of injuries, *μ* is the mean, and *σ* is the standard deviation of the data.

To avoid negative values after normalisation, the linear shift method is continued to eliminate the influence of negative values. The injured area is normalized twice to maintain the data distribution pattern. To address potential redundancy among the three variables—i.e., the more damaged areas there are, the greater the number and area of injuries may be—the Pearson correlation coefficient is used to quantify the correlation among the three variables. If the correlation coefficient r > 0.8, it is considered highly redundant. To avoid redundancy issues among variables, principal component analysis (PCA) is further applied to compress the three redundant variables into a single composite indicator, quantifying the extent of surface damage per fish and establishing damage severity classification criteria.

### 4.8. Histological Analysis

Individual specimens of largemouth bass were selected based on their injury severity, defined as uninjured, minor, moderate, and severe according to the comprehensive injury score. Three largemouth bass were selected from each injury severity group. Two sets of samples were collected from each specimen, including liver, spleen, kidney, muscle, and skin. One set was used for subsequent qRT-PCR quantitative detection, while the other was fixed in Bouin’s fixative for 24 h, After rinsing with 70% ethanol, the samples were stored in 70% ethanol. The tissues were sequentially dehydrated with graded ethanol, cleared with xylene, embedded in paraffin using an automatic embedding machine, and sectioned at a thickness of 7 μm. The sections are then dewaxed with xylene, rehydrated with a gradient ethanol solution, stained with hematoxylin solution for 3–5 min, followed by eosin staining for 5 min, then dehydrated with graded ethanol, washed with xylene, and mounted with neutral balsam. After section preparation, tissue pathological changes are observed using an Eclipse Ci-L microscope.

### 4.9. RNA Extraction, Reverse Transcription, and qRT-PCR Analysis

Following the infection experiment, to further assess the reliability of the comprehensive injury scoring model, qRT-PCR was used to detect the mRNA expression levels of immune-related genes in liver, kidney, spleen, muscle, and skin of largemouth bass infected with LMBV. Total RNA was extracted from liver, kidney, spleen, muscle, and skin of largemouth bass at four different injury severity levels using the VAMNE Magnetic Pathogen RNA Kit (Vazyme, Nanjing, China) and the VNP-96P fully automated nucleic acid extractor. RNA content and purity were measured using 1% agarose gel electrophoresis and NanoDrop spectrophotometry, respectively. RNA was treated with DNase to remove DNA contaminants, and reverse transcription into cDNA was performed using the miRNA 1st Strand cDNA Synthesis Kit. Real-time quantitative PCR was performed using the SYBR^®^ Premix Ex Taq Kit (Takara, Dalian, China) on an ABI QuantStudio 5 instrument (ABI, Foster City, CA, USA). The RT-qPCR reaction mixture was 20 μL, consisting of 10 μL 2× SYBR Premix Ex TaqTM, 1 μL each of forward and reverse primers, 2 μL cDNA template, and RNase-free dH_2_O. The qPCR cycling conditions were as follows: 95 °C for 5 min, followed by 5 cycles at 95 °C, 60 °C for 10 s, and 72 °C for 15 s. Data were calculated using the 2^−ΔΔCt^ method and normalized with β-ACTIN [58]. Each sample was subjected to three independent experiments. The primer sequences used in this study were referenced from published literature [59,60], with specific sequences listed in Table 3.

## 5. Conclusions

In summary, this study successfully established a machine vision-based grading model for LMBV lesions. By integrating three quantitative variables—number of damaged areas, number of lesions, and lesion area—through principal component analysis (PCA), LMBV infection in largemouth bass was classified into four severity levels: undamaged (0), mild (1), moderate (2), and severe (3). Histopathological and qRT-PCR analyses revealed that the severity of external injury correlated with visceral organ pathology and the expression levels of immune genes (e.g., *IL-6*, *TNF-*α). The MV comprehensive injury score effectively reflected the entire process from early local immune activation to late systemic inflammation and immune escape, further validating the model’s reliability. In summary, this study provides scientific evidence for the early prevention and control of largemouth black bass iridovirus, holding significant implications for advancing the intelligent development of aquaculture in the future.

## Figures and Tables

**Table 1:** PCA dimension reduction analysis of three variable data sets.

PCA	Variance Contribution Rate	Cumulative Variance Contribution Rate
PC1	72.30%	72.30%
PC2	21.50%	93.80%
PC3	6.20%	100%

**Table 2:** Principal component loading and interpretation of PC1.

Variable	PC1 Loading	Interpretation
N_1_	0.59	Injury spread range
N_2_	0.63	Injury density
S	0.68	The severe injury of a single region

Note: N_1_ is the number of injury segmentation regions, N_2_ is the number of surface injuries, and S is the area of injuries.

**Table 3:** Primer sequences.

Gene Full Name	Primer Names	Sequence (5′-3′)
*Cytochrome c*	*CYC-F*	AGAAGTGTGCCCAATGCCATACTG
	*CYC-R*	GCGTCCGAACAGACCCCAAAG
*C-X-C motif chemokine ligand 2*	*CXCL2-F*	ACACATTCTGCTGTCCTCGTTTCC
	*CXCL2-R*	TCTACACCCAGGCTCCTCAAACTC
*Suppressor of cytokine signaling 1*	*SOCS1-F*	AGTGTGGTGGTTAGAGATGGGAGAG
	*SOCS1-R*	GAGGATGACGATGATGACGATGAGC
*Interleukin-1 beta*	*IL-Iβ-F*	CGTACATCCGTGCCAACAGI
	*IL-Iβ-R*	ATGCTCTTTAACTCCTCCI
*Tumor necrosis factor-alpha*	*TNF-a-F*	CTAGTGAAGAACCAGATTGT
	*TNF-a-R*	AGGAGACTCTGAACGATG
*Interferon-gamma*	*IFN-Y-F*	TCCCTCTGAAGATGAACAAA
	*IFN-Y-R*	AACGCCACCCATAAACA
*Interleukin-8*	*IL-8-F*	GAGTTTGAGGAGCCTGGGTGT
	*IL-8-R*	GGGTCCAGGCAAACCTCTTG
*Interleukin-6*	*IL-6-F*	GGGAGACTCGCTCTGACCTACTG
	*IL-6-R*	TACCTCCTCCTTGTGGCGTTGG
*Beta-actin (reference gene)*	*β-ACTIN-F*	CCACCACAGCCGAGAGGGAA
	*β-ACTIN-R*	TCATGGTGGATGGGGCCAGG

Experimental data were processed using Excel 2021 and analysed for statistical differences using SPSS v27.0 software. GraphPad Prism v9.5.0 and OriginPro 2024 were used to create graphs, and the data were expressed as mean ± standard deviation (SD). A one-way analysis of variance was used to determine differences, and differences were considered statistically significant when *p* < 0.05.

## Data Availability

All data generated or analyzed during this study are included in this published article and its Supplementary Materials.

## Supplementary Materials

The following supporting information can be downloaded at https://www.mdpi.com/article/10.3390/ijms26188810/s1.
