# Supplementary material for: Development of a Comprehensive Lesion Severity Classification Model for Largemouth Bass (Micropterus salmoides) Ranavirus (LMBV) Based on Machine Vision
Source: Int J Mol Sci. 2025 Sep 10;26(18):8810. doi: 10.3390/ijms26188810 (PMC12469617; doi:10.3390/ijms26188810)
Supplement: Supplementary file 1 [file ijms-26-08810-s001.zip › ijms-3834846-supplementary.pdf]

Table S1 Histological changes at different grades

	liver
Grade 0 (Uninfected)	The hepatic lobules are structurally intact, with hepatocytes exhibiting regular morphology and uniform cytoplasm, showing no necrosis or inflammatory infiltration.
Grade 1 (Mild)	Localized hepatocyte necrosis with cytoplasmic loosening and edema, accompanied by increased staining in some cells.
Grade 2 (Moderate)	Nuclear condensation intensifies, with nuclear fragmentation and dissolution occurring, accompanied by increased inflammatory cell infiltration.
Grade 3 (Severe)	Extensive fibrous tissue proliferation forming pseudo-lobular structures, with hepatocyte clusters being segmented.
	Kidney
Grade 0 (Uninfected)	The glomeruli and tubules are structurally normal, with no abnormalities in the interstitium.
Grade 1 (Mild)	Vacuolar degeneration of renal tubular epithelial cells.
Grade 2 (Moderate)	Increased inflammatory cell infiltration in the stroma, with an increase in the number of granulomas.
Grade 3 (Severe)	Extensive tubular sloughing, tissue necrosis, and increased granulomatous infiltration
	Spleen
Grade 0 (Uninfected)	The splenic corpuscles are clearly defined, with a normal ratio of white pulp to red pulp.
Grade 1 (Mild)	Reduced splenic corpuscles, increased white pulp and lymphocytes, with the presence of a small number of granulomas.

Grade 2 (Moderate)	Multiple granulomas increased, red pulp hyperemia, macrophage aggregation.
Grade 3 (Severe)	Extensive granulomatous inflammation with disruption of splenic tissue architecture and focal necrosis.
	Muscle
Grade 0 (Uninfected)	Muscle fibers are neatly arranged, with no signs of degeneration or inflammation.
Grade 1 (Mild)	Sarcoplasmic vacuolization.
Grade 2 (Moderate)	Inflammatory cell infiltration, increased thickness of the muscle fascia due to edema
Grade 3 (Severe)	Muscle fiber rupture and dissolution, with localized collagen fiber proliferation.
	Skin
Grade 0 (Uninfected)	The collagen fibers in the dermis are loosely arranged, while the epidermis is tightly connected to the dermis.
Grade 1 (Mild)	Hypertrophy of collagen fibers in the dermis, with disappearance of loose connective tissue spaces.
Grade 2 (Moderate)	Edema in the basement membrane zone at the epidermal-dermal junction with appearance of the zona pellucida.
Grade 3 (Severe)	Cytoplasmic vacuolar degeneration, perivascular lymphocyte and macrophage infiltration in the dermis.

Table S2. Summary of Correlations Between MV Injury Grading and Histology, Immunogene Expression

Injury Grade	Representative physical characteristics	Major histopathological changes	Key Immune Gene Expression Trends (Compared to Level 0)
Level 0	No visible injury	The tissue structure is normal, with no signs of inflammation or necrosis.	All investigated genes (IL-6, IL-8, TNF-α, CXCL2, CASP8, CYC) showed baseline expression levels
Level 1	Scattered red hemorrhagic spots (B/E zone)	Localized vacuolar degeneration of hepatocytes; mild inflammatory infiltration in the spleen and kidneys.	Significant upregulation of TNF-α and CXCL2 in liver. SOCS1 significantly upregulated in kidney. CYC peak expression in spleen.
Level 2	Small areas of ulceration; gill covers and caudal peduncle markedly red and swollen.	Multiple granuloma formation; Inflammatory infiltration between muscle fibers; Basement membrane band edema	IL-6 expression increased in spleen vs. Level 0. CXCL2 increased in kidney vs. Level 0. Sustained high expression of apoptosis-related genes.
Level 3	Extensive ulceration and muscle necrosis	Extensive fibrosis of the liver; Large areas of necrosis in the spleen and kidneys; Rupture and dissolution of muscle fibers.	Pro-inflammatory factors (such as IL-6) are persistently overexpressed in the liver, spleen, and kidneys; apoptosis genes (CASP8) show suppressed expression in the spleen.
